# Serial evaluation of liver enzyme activities in dogs with pulmonary coccidioidomycosis administered *per os* fluconazole

**DOI:** 10.3389/fvets.2024.1402572

**Published:** 2024-07-03

**Authors:** Dena Berlin, Jared A. Jaffey, Charlotte Bolch, Tian Zhou, Laura H. Rayhel, Andrew S. Hanzlicek

**Affiliations:** ^1^Department of Specialty Medicine, Midwestern University College of Veterinary Medicine, Glendale, AZ, United States; ^2^Office of Research and Sponsored Programs, Midwestern University, Glendale, AZ, United States; ^3^MiraVista Diagnostics, Indianapolis, IN, United States

**Keywords:** hepatotoxicity, antifungal, valley fever, *Coccidioides*, fluconazole

## Abstract

Pulmonary coccidioidomycosis is a relatively common fungal disorder in dogs that have lived in or traveled to endemic regions and fluconazole is a common antifungal treatment. Liver enzymopathy can occur with fluconazole administration, but the frequency of occurrence nor potentially associative factors have been explored in dogs with pulmonary coccidioidomycosis. Therefore, our objectives were to describe the occurrence and magnitude of liver enzyme activity (LEA) elevation in dogs with pulmonary coccidioidomycosis during treatment with *per os* fluconazole and identify variables associated with liver enzymopathy. This was a retrospective observational study that analyzed serum biochemical data obtained from a separate prospective study that included 32 client-owned dogs with newly diagnosed pulmonary coccidioidomycosis from October 2020 to February 2021. *Per os* fluconazole administration (median dosage: 16.2 mg/kg/day) was initiated after diagnosis and dogs were evaluated once every 3 months thereafter until remission or for a maximum of 12 months. Recorded biochemical parameters at each visit (including baseline) included alanine transaminase (ALT), aspartate aminotransferase (AST), alkaline phosphatase (ALP), and gamma-glutamyl transferase (GGT). Magnitude of increased LEA was based on the fold increase above the upper limit of the reference interval and defined as mild (<5×), moderate (5–10×) or severe (>10×). Forty-seven percent (15/32) of dogs were documented to have elevations in one or more LEAs after initiation of fluconazole administration during the study period. Thirty-four percent and 25% of dogs had elevated ALP and ALT activities, respectively, at some point during treatment. Elevations in AST and GGT activities were rare. The magnitude of LEA elevation was mild in all cases. Logistic regression models did not identify associations between age, weight, sex, neutered status, prednisone administration, fluconazole dose or duration of treatment with the occurrence of liver enzymopathy. Approximately half of dogs with pulmonary coccidioidomycosis are expected to develop mild increases in activities of ALP and/or ALT with rare involvement of AST or GGT at some point during treatment with fluconazole up to 12 months.

## Introduction

Fluconazole is one of the most used antifungals in dogs to treat a variety of systemic mycoses such as coccidioidomycosis, histoplasmosis, blastomycosis, cryptococcosis, aspergillosis (1–5). The primary mechanism of action includes inhibition of ergosterol synthesis, an integral component for fungal cell membrane function (6). The composition of fluconazole imparts several advantages that likely accounts for its frequent use in dogs, including excellent bioavailability that is not altered by food or gastric acidity (6). Moreover, it has lower protein binding, smaller molecular weight, and is more water soluble than other azoles, which improves its diffusion into saliva, urine, synovial fluid, and cerebral spinal fluid (6).

Generally, the adverse effect profile is lower than other azoles like ketoconazole, itraconazole, voriconazole, and posaconazole (6). While uncommon, adverse effects are generally gastrointestinal (e.g., vomiting, diarrhea, alterations to appetite) and transient (1, 2). Fluconazole, like other azole-drugs, can result in hepatocellular damage and increased liver enzyme activity (LEA) that can manifest with overt clinical signs of hepatotoxicosis or can remain entirely subclinical. To the authors’ knowledge, there have only been three published studies that have investigated the hepatotoxic effects of fluconazole, with the evaluation limited predominately to serum alanine aminotransferase (ALT) enzyme activity (1, 2, 7). Those studies did not evaluate other potentially relevant serum LEAs such as alkaline phosphatase (ALP), gamma-glutamyl transferase (GGT), or aspartate aminotransferase (AST) or the expected magnitude of elevation. In addition, the previously mentioned studies did not identify risk factors associated with the occurrence of liver enzymopathy.

*Coccidioides* spp. is endemic to the southwestern United States, Mexico, Central and South America that can cause pulmonary or disseminated disease (8). Fluconazole is typically the first antifungal used to treat coccidioidomycosis in dogs (although no comparative studies have been performed) because of its optimal bioavailability, safety profile, and is less expensive than other azoles used to treat systemic mycoses (8). Anecdotally, mild to moderate increases in serum LEAs are relatively common in dogs with coccidioidomycosis treated with fluconazole, but the frequency and severity of LEA increases has yet to be scientifically evaluated. This information is important because it can contextualize expectations for clinicians managing these dogs with coccidioidomycosis treated with fluconazole.

Therefore, the objective of this study was to determine the frequency, severity, and risk factors for increased LEAs in dogs with pulmonary coccidioidomycosis treated with *per os* fluconazole. We hypothesized that the activity of one or more serum liver enzymes would be increased in ≥50% of dogs with pulmonary coccidioidomycosis treated with fluconazole and that one or more associative variables would be identified.

## Materials and methods

### Criteria for selection of cases

This was a retrospective observational study that examined serum biochemical data from dogs with a new diagnosis of pulmonary coccidioidomycosis enrolled in a separate study with different objectives between October 2020 and February 2021 (9). Written informed consent was obtained for all included dogs. This study was conducted in accordance with guidelines for clinical studies and approved by the Midwestern University Animal Care and Use Committee (protocol # 3024).

Dogs were required to demonstrate at least one clinical sign related to respiratory tract disease including cough, wheeze, increased respiratory effort, exercise intolerance, tachypnea, cyanosis, or syncope in conjunction with one or more positive serum anti-*Coccidioides* antibody serologic test result and had a minimum of two-view thoracic radiographs available for review. Serological testing for immunoglobulin (Ig) M and IgG against *Coccidioides* spp. was performed by agar gel immunodiffusion (AGID) (IgM and IgG) and enzyme immunoassay (EIA; IgG) at a single reference laboratory (MiraVista Diagostics). A positive EIA IgG was defined as results that were ≥10 EIA Units (EU). Positive AGID IgM or IgG was defined as detectable antibodies in an undiluted serum sample. If positive, serial dilutions were tested (up to 1:128) and the highest dilution with detectable IgG antibodies was reported as the final serum titer result. The upper quantifiable range of the EIA IgG was 80 EU. Exclusion criteria included confirmed or suspected disseminated disease, comorbid disorders with potential immune dysregulation (i.e., diabetes mellitus, hyperadrenocorticism, neoplasia etc.), administration of immunomodulating medications, and if antifungal treatment had been initiated for >7 days before enrollment. Prednisone administration at anti-inflammatory dosages for up to 3 weeks after initial diagnosis was permissible.

### Study design

Antifungal therapy was initiated with an FDA-approved generic formulation of fluconazole after diagnosis and dogs were evaluated once every 3 months thereafter until remission was achieved or for a maximum of 12 months. There was no control of manufacturer generic brands of fluconazole administered to dogs. Serial evaluations in all dogs consisted of three-view thoracic radiographs, antibody serologic testing, complete blood count, urinalysis, and a serum biochemical panel. The attending clinicians, not research investigators, made all other diagnostic testing recommendations and treatment decisions. Hematologic and serum biochemical panels and urinalyses were performed at a single veterinary commercial laboratory (Antech Diagnostics) at baseline and at all subsequent visits. Recorded serum biochemical parameters included ALT (reference interval: 12–118 U/L), AST (reference interval: 15–66 U/L), ALP (reference interval: 5–131 U/L), and GGT (reference interval: 1–12 U/L). Magnitude of increased LEA was based on the fold increase of enzyme activity above the upper limit of the reference interval and defined as mild (<5×), moderate (5–10×) or severe (>10×) (10). Other laboratory parameters besides LEAs were not evaluated for this study. Fluconazole administration at the recheck evaluation was required for LEA data to be included in statistical analyses. Alterations in antifungal treatment (i.e., different antifungal medication or compounded formulation) or discontinuation of fluconazole before a scheduled recheck evaluation precluded inclusion of LEA data from that visit and data from the preceding recheck evaluation, at which time the dog had received fluconazole, was retained for analysis.

### Statistical analysis

Statistical analyses were conducted using proprietary software (R version 4.3, R Core Team 2023 and SigmaPlot, Systat Software Inc). Non-normally distributed continuous data was presented as median, interquartile range (IQR), and range when indicated. Normally distributed data was presented as mean and standard deviation (SD). Categorical data were presented as proportions. Logistic regression models were used to assess for associations between relevant baseline variables and the outcome of interest during treatment with *per os* fluconazole. Outcome #1 was the occurrence of an increase in any one of the four liver enzyme parameters (i.e., ALT, ALP, GGT, AST) at any point during treatment with fluconazole. Outcome #2 was the occurrence of an increase in each of the individual liver enzyme parameters separately at any point during treatment. The logistic regression models had the outcome (dependent variable) set up as a dichotomous variable indicating at least one episode of an increased (or not) LEA. The independent variables that were included in each model were age, weight, sex (male or female), neuter status (neutered or intact), prednisone administered (yes or no), fluconazole dose, and duration of fluconazole treatment. The logistic regression model assumptions (i.e., independence of errors, the continuous predictors are linearly related to the logit outcome, absence of multicollinearity, and no influential outliers) were all met even with the smaller sample size of the data. A subset of included dogs at baseline were either administered prednisone for a short period of time and/or had liver enzymopathy detected (*n* = 14). Because these factors have potential to affect LEA after initiation of fluconazole, we performed statistical tests to investigate their effect in our cohort. Mann–Whitney rank sum tests were used to compare LEAs at each evaluation (i.e., 3-month, 6-month, 9-month, and 12-month) after initiation of fluconazole treatment between dogs that were either administered prednisone and/or had a liver enzymopathy (*n* = 14) at baseline and those that were not administered prednisone and had LEA within their respective reference intervals (*n* = 18). A Fisher’s exact test was used to assess whether an association existed between prednisone administration and/or liver enzymopathy at baseline and the occurrence of an increase in any of the liver enzyme parameters at any point during treatment with fluconazole. *p*-values of <0.05 were considered statistically significant.

## Results

### Dogs

**Figure 1:**
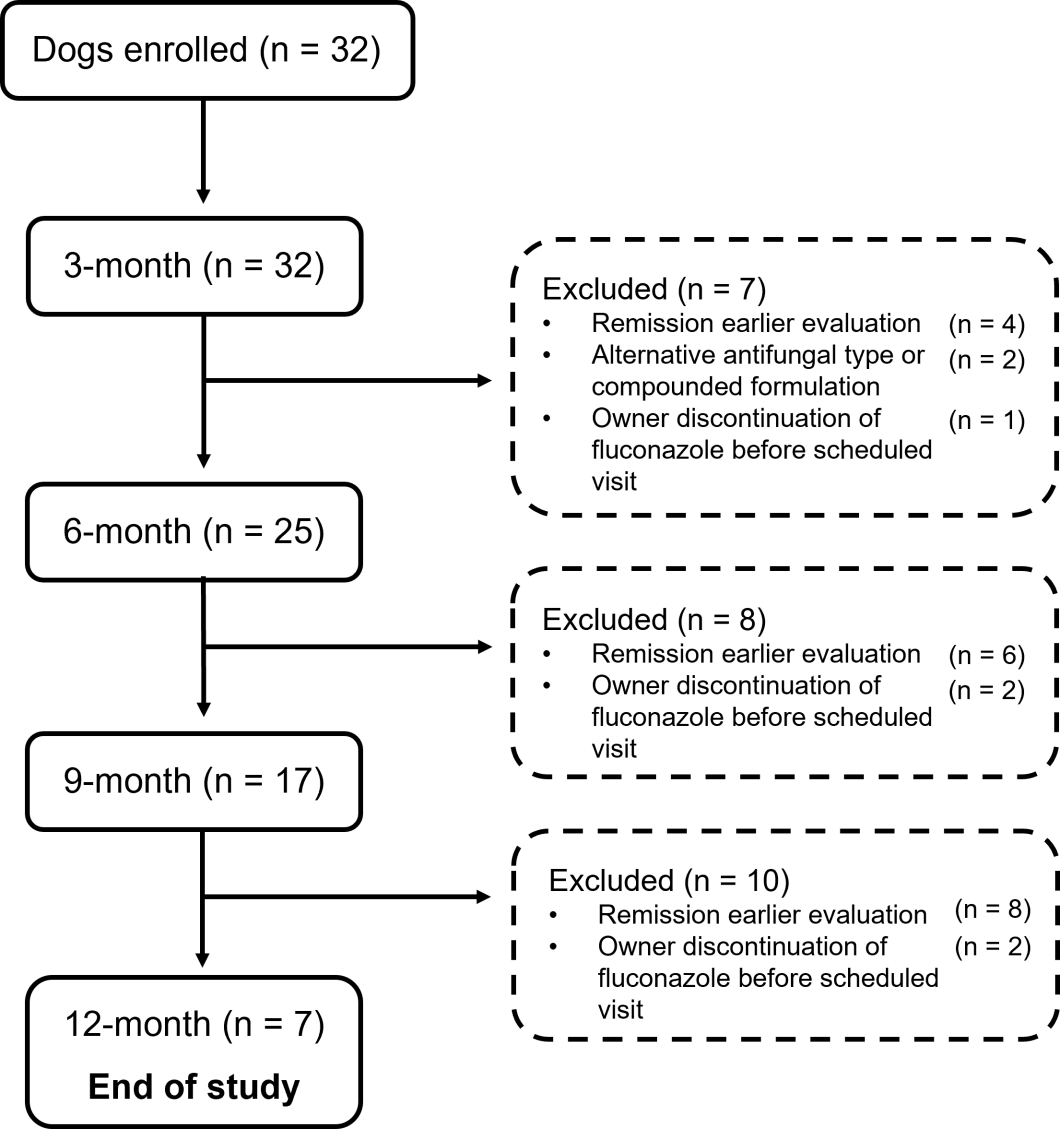
Flow diagram illustrating the number of dogs with newly diagnosed pulmonary coccidioidomycosis enrolled between October 2020 and February 2021 as well as cases available at each recheck examination and number of dogs that were excluded with adjoining reason.

Thirty-two dogs were included in this study (Figure 1). Population characteristics and clinical information for this cohort have been previously reported (9). The median *per os* fluconazole dose prescribed was 16.2 mg/kg/day (IQR, range; 5.3, 10.3–32.3 mg/kg/day). The dose of fluconazole did not change over the course of the study period for dogs that remained on this antifungal. Overall, the median duration of treatment with fluconazole was 275 days (IQR, range; 129.5, 90–459 days). Eight dogs received prednisone after diagnosis, with a mean *per os* dosage of 0.7 mg/kg/day (SD, 0.27) and mean duration of administration of 11.9 days (SD, 3.4). Most of the dogs (63%, 5/8) administered prednisone after diagnosis had normal LEAs throughout the study period (Supplementary Table 1). The remaining three dogs had mild increases in one or more LEAs at some point during the study period (Supplementary Table 1). Twenty-eight percent (9/32) of dogs had increased LEAs at baseline with additional details outlined below. No dogs were administered liver supportive supplements.

Fifty-six percent (18/32) of dogs had unremarkable baseline LEAs and were not administered prednisone. The median *per os* fluconazole dose prescribed to these dogs was 14.5 mg/kg/day (IQR, range; 5.3, 10.3–32.3 mg/kg/day). The median duration of treatment with fluconazole was 284.5 days (IQR, range; 225.8, 90–446 days).

### Baseline evaluation

One dog had a presumed subclinical liver enzymopathy secondary to empiric administration of doxycycline preceding the diagnosis of pulmonary coccidioidomycosis that resolved shortly after discontinuation of the offending drug. The LEAs in this dog were as follows: ALT: 7,278 U/L, AST: 1,658 U/L, ALP: 3,363 U/L, and GGT: 105 U/L. There were no other dogs with increased ALT or AST activities at the baseline evaluation. Twenty-eight percent (9/32) of dogs, including the dog with doxycycline mediated liver enzymopathy, had an increased ALP activity of which the median was 234 U/L (IQR, range; 161.5, 133–3,363 U/L) (Table 1 and Supplementary Table 1). The magnitude of ALP activity increase was mild in all dogs (89%, *n* = 8) except the dog with doxycycline related hepatotoxicity. Six percent of dogs (2/32) had an increased GGT activity of which the magnitude of increase was categorized as one each of moderate (doxycycline dog; 105 U/L) and mild (13 U/L) (Table 1).

**Table 1:** Descriptive summary of alanine aminotransferase (ALT), alkaline phosphatase (ALP), aspartate aminotransferase (AST), and gamma-glutamyl transferase (GGT) activities in dogs with newly diagnosed pulmonary coccidioidomycosis before (baseline) and 3 months, 6 months, 9 months, and 12 months after initiation of *per os* fluconazole administration.

Variable	Baseline (*n* = 32)	3-month (*n* = 32)	6-month (*n* = 25)	9-month (*n* = 17)	12-month (*n* = 7)	Reference interval (U/L)
Duration of fluconazole administration (days)	—	93.5 (6.8, 71–110)	187 (17.5, 174–229)	294 (22.5, 275–333)	397 (62, 375–459)	—
ALT (U/L)	27.5 (24.8, 8–7,270)	52 (30.5, 19–241)	59 (54.5, 21–148)	64 (52.5, 33–196)	69 (85, 34–128)	12–118
AST (U/L)	30 (10.3, 18–1,658)	31.5 (16.8, 16–58)	29 (12, 21–85)	29 (19.5, 17–69)	22 (8, 21–37)	15–66
ALP (U/L)	87.5 (82.3, 18–3,363)	88.5 (141.5, 21–403)	73 (44.5, 15–421)	79 (105, 19–495)	92 (105, 27–418)	5–131
GGT (U/L)	3 (1.8, 1–105)	5 (4, 2–31)	4 (2.5, 1–12)	4 (2.5, 2–8)	4 (3, 1–8)	1–12

Data presented as median (interquartile range and range of data).

The median (IQR and range) LEAs for the 18 dogs not administered prednisone and with results within their respective reference intervals were ALT: 27 U/L (23.8, 11–85 U/L), ALP: 73 U/L (66.3, 18–120 U/L), GGT: 3 U/L (2, 1–7 U/L), and AST: 29 U/L (9, 18–47 U/L).

### 3-month evaluation

**Figure 2:**
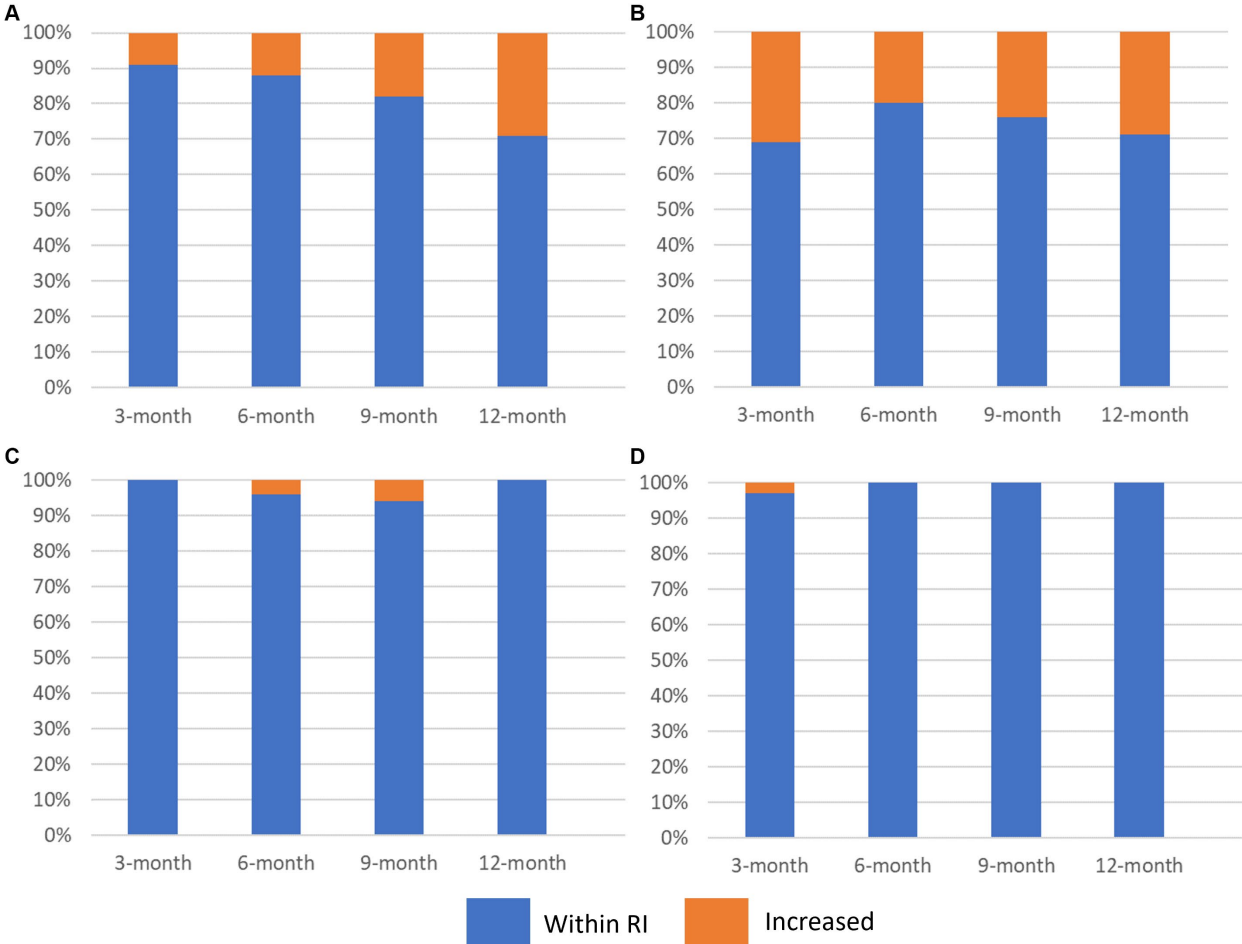
Stacked bar graph (100%) illustrating the proportion of dogs with pulmonary coccidioidomycosis that had increased activities of **(A)** alanine aminotransferase [ALT], **(B)** alkaline phosphatase [ALP], **(C)** aspartate aminotransferase [AST], and **(D)** gamma-glutamyl transferase [GGT] 3 months, 6 months, 9 months, and 12 months after initiation of *per os* fluconazole. Blue represents the proportion of dogs with the liver enzyme parameter within the reference interval and orange represents dogs with liver enzyme activities above the upper limit of the reference interval.

Thirty-two dogs were examined at the 3-month evaluation and the median duration of fluconazole administration was 93.5 days (IQR, range; 6.8, 72–110 days) (Figure 1). The dog with doxycycline related hepatotoxicity had LEAs within their respective reference intervals. The median LEAs were ALT: 52 U/L, AST: 31.5 U/L, ALP: 88.5 U/L, and GGT: 5 U/L (Table 1). Thirty-four percent (11/32) of dogs had an increase in ≥1 LEA (Figure 2). Of these dogs, most (82%, 9/11) had an increase in a single LEA. One dog had increases in ALT and ALP activities and the other dog had increases in ALT, ALP, and GGT activities. The most frequently increased LEA was ALP (91%, 10/11). Increased activities of ALT and GGT were identified in 27% (3/11) and 9% (1/11) of dogs, respectively. No dog had an increase in AST activity. Of the dogs with increased ALP activity, the median was 234 U/L (IQR, range; 70.5, 147–403 U/L). Similarly, the median ALT activity of dogs with increases was 215 U/L (IQR, range; 115, 126–241 U/L). The GGT activity in the one dog in which it was increased was 31 U/L. The magnitude of increased LEAs was mild in all dogs.

Twenty-eight percent (5/18) of dogs not administered prednisone and that had unremarkable LEAs at baseline had an increase in ≥1 LEA. Most of these dogs (80%, 4/5) had an increase in a single LEA. All five dogs had mild elevations in ALP activity and one dog had a concurrent mild increase in ALT activity. The median (IQR and range) LEAs were ALT: 51 U/L (30.8, 19–215 U/L), AST: 32 U/L (16.5, 16–58 U/L), ALP: 75.5 U/L (175.5, 21–301 U/L), and GGT: 5 U/L (4, 1–8 U/L).

### 6-month evaluation

Twenty-five dogs had data available at the 6-month evaluation (Figure 1). The median duration of fluconazole administration was 187 days (IQR, range; 17.5, 174–229 days). The median LEAs were ALT: 59 U/L, AST: 29 U/L, ALP: 73 U/L, and GGT: 4 U/L (Table 1). Twenty-four percent (6/24) of dogs had an increase in ≥1 LEA and most dogs (67%, 4/6; Figure 2) had an increase in a single LEA. One dog had increases in ALT and ALP activities and the other dog had increases in ALT, ALP, and AST activities. Alkaline phosphatase was the most commonly increased LEA (83%, 5/6) followed by ALT (50%, 3/6). Aspartate aminotransferase activity was increased in one dog (85 U/L) and no dog had an increased GGT activity. The median ALP and ALT activities in dogs with increased results were 370 U/L (IQR, range; 170.5, 225–421 U/L) and 138 U/L (IQR, range; 26, 122–148 U/L), respectively. The magnitude of increased LEAs was mild in all dogs.

Thirteen of the original 18 dogs not administered prednisone and with unremarkable baseline LEAs had data available at the 6-month evaluation. A single dog had a mild elevation in ALT and ALP activities. Therefore, 92% (12/13) had LEAs within their respective reference intervals. The median (IQR and range) LEAs were ALT: 47 U/L (53.5, 32–148 U/L), AST: 32 U/L (15.5, 21–54 U/L), ALP: 70 U/L (52.5, 15–370 U/L), and GGT: 4 U/L (5, 1–12 U/L).

### 9-month evaluation

Seventeen dogs had data available from the 9-month evaluation (Figure 1). The median duration of fluconazole administration was 294 days (IQR, range; 22.5, 275–333 days). The median LEAs were ALT: 64 U/L, AST: 29 U/L, ALP: 79 U/L, and GGT: 4 U/L (Table 1). Thirty-five percent (6/17) of dogs had an increase in ≥1 LEA (Figure 2). Most of these dogs (67%, 4/6) had an increase in a single LEA. One dog had increases in ALT and ALP activities and the other dog had increases in ALT and AST activities. The most frequently increased LEA was ALP (67%, 4/6) followed by ALT (50%, 3/6). Aspartate aminotransferase activity was increased in one dog (69 U/L) and no dog had an increased GGT activity. The median ALP and ALT activities in dogs with increased results were 221 U/L (IQR, range; 235.8, 181–495 U/L) and 150 U/L (IQR, range; 52, 144–196 U/L), respectively. The magnitude of increased LEAs was mild in all dogs.

Ten of the original 18 dogs not administered prednisone and with unremarkable baseline LEAs had data available at the 9-month evaluation. Forty percent (4/10) of dogs had an increase in ≥1 LEA. Two dogs each had increases in two LEAs. One dog had mild elevations in ALP and ALT while the other dog had mild elevations in ALT and AST activities. The remaining two dogs had a mild increase in ALP and ALT activity, respectively. The median (IQR and range) LEAs were ALT: 61 U/L (97.5, 39–196 U/L), AST: 32.5 U/L (25.8, 18–69 U/L), ALP: 78 U/L (106.3, 19–495 U/L), and GGT: 4 U/L (3.5, 2–8 U/L).

### 12-month evaluation

Seven dogs had data available from the 12-month evaluation (Figure 1). The median duration of fluconazole administration was 397 days (IQR, range; 62, 375–459 days). The median LEAs were ALT: 69 U/L, AST: 22 U/L, ALP: 92 U/L, and GGT: 4 U/L (Table 1). Forty-three percent (3/7) of dogs had an increase in ≥1 LEA (Figure 2). One dog had mild increases in both ALT (120 U/L) and ALP (418 U/L) activities and the remaining two dogs had singular mild elevations in ALT (128 U/L) and ALP (136 U/L), respectively. No dogs had increased AST or GGT activities.

Four of the original 18 dogs not administered prednisone and with unremarkable baseline LEAs had data available at the 12-month evaluation. A single dog had a mild increase in ALP activity. The remaining dogs had LEAs within their respective reference intervals. The median (IQR and range) LEAs were ALT: 55.5 U/L (46.5, 34–87 U/L), AST: 22 U/L (2.3, 21–24 U/L), ALP: 84 U/L (82.8, 31–136 U/L) and GGT: 4.5 U/L (2.5, 2–5 U/L).

### Temporal assessment of liver enzyme activities

Fifty-six percent (5/9) of the dogs with an increase in ≥1 LEA at baseline had resolution of liver enzymopathy at the subsequent evaluation after being treated with fluconazole for approximately 3 months. Of the four dogs with persistently increased LEAs at the 3-month evaluation, two dogs had persistence of mild ALP activity elevations. One dog originally had a mild increase in ALP activity that changed to a mildly increased ALT activity. The remaining dog initially had mild elevations in ALP and GGT activities with subsequent persistence of only increased ALP activity.

Forty-seven percent (15/32) of dogs were documented to have elevations in one or more liver enzyme parameters after initiation of fluconazole administration during the study period. Twenty-six percent (4/15) of these dogs had a liver enzymopathy at baseline. Overall, 13% (4/32) of dogs that had a liver enzymopathy at baseline had one or more subsequent visits with one or more LEA elevations. Twenty-five percent (8/32) and 34% (11/32) of dogs experienced at least one episode of increased ALT or ALP activity, respectively, at some point during treatment. Liver enzyme activity elevations were first identified most commonly at the 3-month evaluation (73%, 11/15). First time identification of liver enzymopathy occurred infrequently at later visits: 6-month (6%, 1/15), 9-month (13%, 2/15), and 12-month (6%, 1/15). Sixty-seven percent (10/15) of the dogs with ≥1 liver enzyme elevation had one or more subsequent scheduled recheck evaluations to assess temporal changes. Liver enzymopathy was identified on ≥1 subsequent evaluation in 70% (7/10) of those dogs but the magnitude remained mild in all cases. Temporal changes in LEAs for individual dogs can be found in Supplementary Table 1.

Forty-four percent (8/18) of dogs that were not administered prednisone and had unremarkable LEAs at baseline were documented to have elevations in one or more liver enzyme parameters after initiation of fluconazole administration during the study period. Thirty-nine percent (7/18) and 22% (4/18) of dogs had at least one episode of increased ALP and ALT activity, respectively, at some point during treatment. Liver enzyme activity elevations were first identified most commonly at the 3-month evaluation (63%, 5/8). First time identification of liver enzymopathy occurred uncommonly at later visits: 9-month (11%, 2/18), and 12-month (6%, 1/18).

While not an objective of this study, it is important to note that no dogs developed hyperbilirubinemia or clinical signs associated with hepatotoxicity during the study period.

### Effect of baseline prednisone administration and liver enzymopathy

There was no difference in ALT, ALP, GGT, or AST activities at any time point after initiation of fluconazole treatment between dogs that were either administered prednisone or had a liver enzymopathy at baseline and those that were not administered prednisone and had LEA within their respective reference intervals (data not shown). There was no difference in the occurrence of one or more increased liver enzyme parameters after initiation of fluconazole administration between dogs that were either administered prednisone or had a liver enzymopathy at baseline and those that were not administered prednisone and had LEA within their respective reference intervals (data not shown).

### Variables associated with increased liver enzyme activities

First, we were interested in whether there were any risk factors at baseline that were associated with the development of an elevation in any of the four liver enzyme parameters (i.e., ALT, ALP, AST, or GGT) at any point during treatment with *per os* fluconazole. There was no association between any of the investigated variables and the occurrence of a liver enzymopathy (Table 2). Next, similar analyses were performed but the goal was to assess whether there were any risk factors for the development of an elevation in each individual liver enzyme parameter during the study period. Association statistics were not performed for AST and GGT because there were so few episodes with increased activities. Similar to the overall analysis, there were no associations identified with the occurrence of elevations in either ALT or ALP activities (Supplementary Tables 2, 3).

**Table 2:** Association between select baseline variables and the occurrence of an increase in any one of the four liver enzyme parameters (i.e., ALT, ALP, GGT, AST) at any point during treatment with *per os* fluconazole in 32 dogs with pulmonary coccidioidomycosis.

Variable	Odds ratio	95% CI	*p*-value
**Age (year)**	1.15	0.89–1.51	0.3
**Weight (kg)**	0.97	0.88–1.05	0.4
**Sex**			
Male	—	—	
Female	1.41	0.23–10.6	0.7
**Neutered**			
Intact	—	—	
Neutered	0.93	0.12–7.55	>0.9
**Prednisone administration**			
No prednisone	—	—	
Prednisone	0.34	0.04–2.25	0.3
**Fluconazole dose (mg/kg/day)**	1.07	0.86–1.41	0.6
**Duration of fluconazole administration (days)**	1.00	1.00–1.01	0.3

Kg, kilogram; CI, confidence interval; mg, milligram.

## Discussion

This retrospective cohort study investigated the occurrence and risk factors for the development of increased LEAs in dogs with pulmonary coccidioidomycosis treated with *per os* fluconazole. We found that approximately half of the dogs in our cohort developed a mild liver enzymopathy at some point during treatment, regardless of whether they were administered prednisone or had a liver enzymopathy at baseline. Most episodes of increased LEAs were characterized by a single abnormal liver enzyme parameter with ALP most frequently identified. Lastly, no associations were identified between baseline variables and the occurrence of liver enzymopathy.

Forty-seven percent of dogs in our cohort developed a mild liver enzymopathy at some point during treatment with *per os* fluconazole. A similar frequency (44%) was identified in dogs that were not administered prednisone and had LEAs within their respective reference at baseline. These results are consistent with the authors’ clinical experience. There is limited information in the literature related to hepatic injury and liver enzymopathy in dogs treated long-term with *per os* fluconazole (1, 2, 7). The inclusion of drug-induced liver injury data in these studies was a byproduct of including a broad description of adverse events presumably associated with fluconazole administration in dogs and not solely focused on liver injury. As such, the scope of assessments was limited predominately to the incidence, and occasionally, severity of ALT activity elevations. One retrospective study found that 17% (3/18) of dogs administered *per os* fluconazole for a median of 183 days (range, 73–296 days) developed a mildly increased ALT activity at some point during treatment for blastomycosis (2). Another retrospective study found that 32.7% (16/49) of dogs with coccidioidomycosis developed an increased ALT activity during the course of treatment with *per os* fluconazole and the median duration of administration was 298.5 days (range, 130–1,000 days) (7). The prevalence of increased ALT activity and duration of treatment in these studies are comparable to our results in which the median duration of treatment was 275 days (range, 90–459 days) and 25% of dogs had an increased ALT activity at some point during treatment.

Understanding the effects that long-term *per os* fluconazole administration has on ALT activity is valuable information but is just one puzzle piece for the clinician when evaluating a serum biochemical profile in dogs during the course of treatment. Results from our study provide clinicians with a more comprehensive assessment of LEAs and a general idea of what changes to expect, which can influence clinical decisions. Thirty-five percent of dogs had an increased ALP activity after initiation of *per os* fluconazole at some point during treatment, making it the most common abnormal liver enzyme parameter in our study while increased AST (two episodes) and GGT (one episode) activities were rare. A similar frequency (39%) of increased ALP activity was identified in dogs that were not administered prednisone and had LEAs within their respective reference intervals at baseline. Collectively, these results suggest that clinicians can expect that if a dog with pulmonary coccidioidomycosis develops increased LEAs during treatment with fluconazole that there will likely be either a mild cholestatic or mixed pattern that does not usually involve AST or GGT. However, while not identified in our sample population, in the authors’ experience, more severe liver enzymopathies with or without clinical signs associated with hepatotoxicity are occasionally identified in dogs with fluconazole administration.

A large meta-analysis of antifungal usage in humans found that the risk for liver injury with standard doses of fluconazole was uncommon with an occurrence of 9.3% (11). The Wang et al. study also reported that discontinuation of fluconazole because of liver injury occurred in only 0.7% of patients (11). Interestingly, liver enzyme elevations in humans treated with fluconazole are usually transient and can resolve despite continued administration (12). Similar to results from our study, the pattern of liver enzymopathy in humans treated with fluconazole is usually cholestatic or mixed (12). Overall, drug-induced liver injury related to long-term fluconazole administration is uncommon in dogs and humans but still has the potential to occur and thus should be monitored with serial serum biochemical examinations.

We did not identify any baseline risk factors associated with the development of increased LEAs in our study. Our results suggest that the investigated variables may have no relevant bearing on the development of liver enzymopathy in dogs with pulmonary coccidioidomycosis treated with *per os* fluconazole. The lack of association with fluconazole dose with increased LEAs was unexpected. There is limited information in humans and rats that suggest fluconazole related liver injury is dose-dependent (13–15). Despite our results, the authors’ clinical impression is that this is also likely true in dogs. Anecdotally, fluconazole dose reductions generally improve or resolve liver enzymopathies in dogs. One possible reason for the lack of association is there was a relatively narrow range of doses administered to dogs in our study. Another potential explanation is that the delivered *per os* dose of fluconazole may not accurately reflect serum drug concentrations, which is likely more important when considering toxicity, because of inter-dog variability in absorption and metabolism (1). There is limited information whether there is an association between duration of fluconazole administration and liver injury (12, 16). A systematic review in pediatric patients found no association between duration of fluconazole administration and the occurrence of liver injury (16). The small sample size may have also impacted our ability to detect associations (i.e., type 2 error). It is also important to note that given the retrospective design of this study and the relatively small sample population there were many potentially important variables not accounted for that can contribute to the development of liver enzymopathy with fluconazole treatment. One example is not knowing whether dogs had a history of clinically relevant liver disease. This information was not exclusionary for dogs in the primary study and therefore this information was not explored or available but may have impacted our objective of describing the effects of fluconazole on LEAs. One study showed that people with pre-existing liver disease had a higher risk of liver injury with fluconazole administration compared to patients without (17). Other variables our study did not account for that may be important for the population of dogs at large, include presence of comorbid disorders, drug–drug interactions, and genetic factors. Additional research is needed with a more expansive sample population that also accounts for the aforementioned variables to better understand associative factors related to the development of liver enzymopathy in dogs with pulmonary coccidioidomycosis treated with fluconazole.

Our study had several limitations that should be considered. Many of the limitations are related to the fact that this was a retrospective examination of data retrieved from a separate study and not a stand-alone investigation. Specifically, this cohort was used primarily in a different study that investigated other objectives and thus some variables were not controlled for because of the link in study design. One example is the lack of serum fluconazole concentrations, which would have allowed a more direct assessment of fluconazole and liver injury. There were nine dogs (28%) in this study that had increased LEAs at baseline before initiation of fluconazole administration. These elevations were almost exclusively a singular mild elevation in ALP activity with the exception of the dog with subclinical doxycycline mediated hepatotoxicity. The specific cause for the mild elevation in ALP activity in the remaining eight dogs is unknown. More than half of the dogs with one or more elevated LEAs at baseline had resolution at all subsequent visits after initiation of fluconazole. This suggests there may have been unaccounted circumstances at baseline that caused increased ALP activity that subsequently resolved. One possibility is induction of ALP enzyme related to stress associated with acute or chronic illness (18). There is limited information on liver enzymopathies in dogs with pulmonary coccidioidomycosis at the time of diagnosis; however, a study in people reported 16% of patients had increases in one or more LEAs at baseline (19). The persistence in mild liver enzymopathy beyond baseline in four dogs may have been related to pre-existing liver disease, fluconazole administration, an unknown cause, or a combination thereof. The nine dogs with increased LEAs at baseline were included in our study for the following reasons. First, more than half of the dogs had subsequent resolution of LEAs at all subsequent examinations despite continued fluconazole administration. Secondly, these results provide practical and clinically relevant information on expectations in LEA changes in dogs treated with fluconazole over time that had a liver enzymopathy at the time of diagnosis.

Another limitation is that eight dogs were administered anti-inflammatory doses of prednisone for a short duration after diagnosis, which may have affected LEAs at the 3-month visit. It is unclear whether the dose and short duration of administration would have resulted in sustained LEA elevations recorded at the 3-month evaluation. Most of the dogs (63%; 5/8) administered prednisone had unremarkable LEAs at all subsequent recheck visits and there was no association between prednisone administration and the occurrence of liver enzymopathy in our study. Dogs administered prednisone after diagnosis was permissible because it is a common practice by veterinarians to mitigate clinical signs after diagnosis and understanding its influence on LEAs in conjunction with fluconazole administration has clinical value. Overall, there were no differences in LEAs at any time point or the frequency of liver enzymopathy after initiation of fluconazole treatment between dogs administered prednisone and/or that had a liver enzymopathy at baseline. Dogs were treated with FDA-approved generic formulations of fluconazole, but the manufacturer was not controlled. A recent study highlighted that not all FDA approved generic formulations of fluconazole are bioequivalent in dogs, as they are in humans (20). Therefore, future studies aimed at replicating our study objectives should prioritize a single formulation of fluconazole and/or measure serum fluconazole concentrations. Lastly, we cannot say with any certainty that long-term fluconazole administration caused liver injury or was the primary cause of liver enzymopathy because liver biopsies were not retrieved before and after dogs received fluconazole. Despite the limitations of this study, our results provide practical information that will guide clinicians managing dogs with pulmonary coccidioidomycosis treated with fluconazole.

## Conclusion

In conclusion, this study found that approximately half of dogs with pulmonary coccidioidomycosis developed mild increases in one or more liver enzyme parameters at some point during treatment with *per os* fluconazole up to 12 months. The biochemical pattern was mostly cholestatic or mixed with rare elevations in AST or GGT activities. Lastly, variables including age, sex, neutered status, prednisone administration, fluconazole dose and duration of administration were not associated with the occurrence of liver enzymopathy.

## Data availability statement

The original contributions presented in the study are included in the article/Supplementary material, further inquiries can be directed to the corresponding author.

## Ethics statement

The animal studies were approved by the Midwestern University Animal Care and Sue Committee (protocol #3024) with written owner consent. The studies were conducted in accordance with the local legislation and institutional requirements. Written informed consent was obtained from the owners for the participation of their animals in this study.

## Author contributions

DB: Data curation, Writing – original draft, Writing – review & editing. JJ: Conceptualization, Data curation, Formal analysis, Investigation, Methodology, Writing – original draft, Writing – review & editing. CB: Formal analysis, Methodology, Writing – original draft, Writing – review & editing. TZ: Formal analysis, Writing – original draft, Writing – review & editing. LR: Funding acquisition, Writing – original draft, Writing – review & editing. AH: Conceptualization, Funding acquisition, Investigation, Writing – original draft, Writing – review & editing.
