# Supplementary material for: Serial evaluation of liver enzyme activities in dogs with pulmonary coccidioidomycosis administered per os fluconazole
Source: Front Vet Sci. 2024 Jul 3;11:1402572. doi: 10.3389/fvets.2024.1402572 (PMC11417468; doi:10.3389/fvets.2024.1402572)
Supplement: Supplementary file 1 [file Table_1.DOCX]

**Supplemental Table 1.** Temporal changes in liver enzyme activities in individual dogs with pulmonary coccidioidomycosis before (baseline) and 3 months, 6 months, 9 months, and 12 months after initiation of per os fluconazole administration. Bolded values indicate results above the upper limit of the reference interval.

| **Dog #** | **Prednisone** | **Visit** | **ALT (U/L)**  **(12-118)** | **AST (U/L)**  **(15-66)** | **ALP (U/L)**  **(5-131)** | **GGT (U/L)**  **(1-12)** |
| --- | --- | --- | --- | --- | --- | --- |
| Dog 1 | Yes | Baseline | 29 | 31 | **343** | 3 |
|  |  | 3-month | 23 | 26 | 97 | 3 |
|  |  | 6-month | --- | --- | --- | --- |
|  |  | 9-month | --- | --- | --- | --- |
|  |  | 12-month | --- | --- | --- | --- |
| Dog 2 | No | Baseline | 29 | 31 | 33 | 2 |
|  |  | 3-month | 46 | 32 | 35 | 3 |
|  |  | 6-month | 46 | 28 | 15 | 5 |
|  |  | 9-month | 64 | 40 | 19 | 2 |
|  |  | 12-month | --- | --- | --- | --- |
| Dog 3 | No | Baseline | 8 | 40 | **266** | 3 |
|  |  | 3-month | **126** | 46 | 85 | 8 |
|  |  | 6-month | **138** | **85** | **236** | 4 |
|  |  | 9-month | --- | --- | --- | --- |
|  |  | 12-month | --- | --- | --- | --- |
| Dog 4 | No | Baseline | 85 | 24 | 44 | 3 |
|  |  | 3-month | 60 | 16 | 119 | 8 |
|  |  | 6-month | 51 | 22 | 92 | 2 |
|  |  | 9-month | 48 | 25 | 131 | 3 |
|  |  | 12-month | 69 | 22 | 92 | 4 |
| Dog 5 | No | Baseline | 27 | 32 | 88 | 2 |
|  |  | 3-month | 19 | 26 | 65 | 3 |
|  |  | 6-month | 40 | 33 | 55 | 4 |
|  |  | 9-month | --- | --- | --- | --- |
|  |  | 12-month | --- | --- | --- | --- |
| Dog 6 | No | Baseline | 27 | 32 | 18 | 3 |
|  |  | 3-month | 59 | 34 | 21 | 2 |
|  |  | 6-month | 39 | 26 | 15 | 3 |
|  |  | 9-month | --- | --- | --- | --- |
|  |  | 12-month | --- | --- | --- | --- |
| Dog 7 | No | Baseline | 28 | 23 | 61 | 4 |
|  |  | 3-month | 63 | 52 | 72 | 5 |
|  |  | 6-month | 79 | 45 | 35 | 5 |
|  |  | 9-month | 53 | 31 | 39 | 8 |
|  |  | 12-month | --- | --- | --- | --- |
| Dog 8 | No | Baseline | **7278** | **1658** | **3363** | **105** |
|  |  | 3-month | 39 | 27 | 25 | 5 |
|  |  | 6-month | --- | --- | --- | --- |
|  |  | 9-month | --- | --- | --- | --- |
|  |  | 12-month | --- | --- | --- | --- |
| Dog 9 | No | Baseline | 99 | 19 | **250** | 4 |
|  |  | 3-month | 57 | 27 | **151** | 7 |
|  |  | 6-month | 83 | 26 | **381** | 2 |
|  |  | 9-month | 60 | 17 | **222** | 6 |
|  |  | 12-month | **120** | 22 | **418** | 2 |
| Dog 10 | No | Baseline | 48 | 28 | 87 | 2 |
|  |  | 3-month | 73 | 32 | **269** | 6 |
|  |  | 6-month | **148** | 35 | **370** | 12 |
|  |  | 9-month | **144** | 34 | **495** | 8 |
|  |  | 12-month | --- | --- | --- | --- |
| Dog 11 | Yes | Baseline | 39 | 36 | 76 | 2 |
|  |  | 3-month | 90 | 44 | 80 | 2 |
|  |  | 6-month | **122** | 50 | 64 | 4 |
|  |  | 9-month | 116 | 49 | 60 | 3 |
|  |  | 12-month | --- | --- | --- | --- |
| Dog 12 | Yes | Baseline | 15 | 31 | **149** | 3 |
|  |  | 3-month | 36 | 22 | 77 | 4 |
|  |  | 6-month | 21 | 25 | 74 | 5 |
|  |  | 9-month | 81 | 31 | 71 | 4 |
|  |  | 12-month | --- | --- | --- | --- |
| Dog 13 | No | Baseline | 46 | 23 | 110 | 4 |
|  |  | 3-month | 56 | 24 | 109 | 5 |
|  |  | 6-month | 42 | 21 | 82 | 4 |
|  |  | 9-month | 67 | 18 | **181** | 6 |
|  |  | 12-month | 42 | 22 | 76 | 5 |
| Dog 14 | No | Baseline | 16 | 29 | 120 | 4 |
|  |  | 3-month | 31 | 22 | 92 | 7 |
|  |  | 6-month | 47 | 26 | 96 | 9 |
|  |  | 9-month | 48 | 23 | 106 | 4 |
|  |  | 12-month | 87 | 24 | **136** | 5 |
| Dog 15 | Yes | Baseline | 23 | 30 | 52 | 3 |
|  |  | 3-month | 54 | 31 | 66 | 4 |
|  |  | 6-month | 72 | 29 | 66 | 4 |
|  |  | 9-month | --- | --- | --- | --- |
|  |  | 12-month | --- | --- | --- | --- |
| Dog 16 | Yes | Baseline | 23 | 27 | 73 | 5 |
|  |  | 3-month | **241** | 36 | **232** | **31** |
|  |  | 6-month | 48 | 23 | 95 | 3 |
|  |  | 9-month | 85 | 29 | 107 | 5 |
|  |  | 12-month | **128** | 37 | 107 | 8 |
| Dog 17 | Yes | Baseline | 31 | 29 | 59 | 4 |
|  |  | 3-month | 29 | 23 | **254** | 2 |
|  |  | 6-month | 39 | 25 | **421** | 1 |
|  |  | 9-month | 43 | 23 | **221** | 3 |
|  |  | 12-month | --- | --- | --- | --- |
| Dog 18 | No | Baseline | 25 | 47 | 33 | 3 |
|  |  | 3-month | 46 | 43 | 68 | 7 |
|  |  | 6-month | 107 | 54 | 77 | 8 |
|  |  | 9-month | 58 | 45 | 42 | 3 |
|  |  | 12-month | --- | --- | --- | --- |
| Dog 19 | No | Baseline | 11 | 18 | 87 | 3 |
|  |  | 3-month | 34 | 32 | 56 | 3 |
|  |  | 6-month | 37 | 38 | 34 | 1 |
|  |  | 9-month | 39 | 23 | 32 | 4 |
|  |  | 12-month | 34 | 21 | 31 | 2 |
| Dog 20 | No | Baseline | 39 | 66 | **158** | **13** |
|  |  | 3-month | 53 | 30 | **403** | 9 |
|  |  | 6-month | 59 | 29 | **225** | 4 |
|  |  | 9-month | --- | --- | --- | --- |
|  |  | 12-month | --- | --- | --- | --- |
| Dog 21 | No | Baseline | 26 | 42 | 23 | 7 |
|  |  | 3-month | 63 | 42 | 65 | 7 |
|  |  | 6-month | 114 | 49 | 72 | 7 |
|  |  | 9-month | **150** | 60 | 64 | 4 |
|  |  | 12-month | --- | --- | --- | --- |
| Dog 22 | No | Baseline | 43 | 30 | 77 | 2 |
|  |  | 3-month | **215** | 58 | **301** | 2 |
|  |  | 6-month | --- | --- | --- | --- |
|  |  | 9-month | --- | --- | --- | --- |
|  |  | 12-month | --- | --- | --- | --- |
| Dog 23 | No | Baseline | 16 | 20 | **133** | 5 |
|  |  | 3-month | 32 | 27 | **147** | 2 |
|  |  | 6-month | 73 | 38 | 112 | 5 |
|  |  | 9-month | 80 | 29 | 79 | 5 |
|  |  | 12-month | --- | --- | --- | --- |
| Dog 24 | No | Baseline | 55 | 35 | 69 | 5 |
|  |  | 3-month | 84 | 45 | 79 | 7 |
|  |  | 6-month | --- | --- | --- | --- |
|  |  | 9-month | --- | --- | --- | --- |
|  |  | 12-month | --- | --- | --- | --- |
| Dog 25 | No | Baseline | 20 | 29 | 106 | 1 |
|  |  | 3-month | 33 | 26 | **225** | 6 |
|  |  | 6-month | 79 | 32 | 68 | 4 |
|  |  | 9-month | **196** | **69** | 92 | 3 |
|  |  | 12-month | --- | --- | --- | --- |
| Dog 26 | Yes | Baseline | 48 | 30 | 96 | 4 |
|  |  | 3-month | 64 | 24 | 47 | 8 |
|  |  | 6-month | 109 | 27 | 16 | 5 |
|  |  | 9-month | --- | --- | --- | --- |
|  |  | 12-month | --- | --- | --- | --- |
| Dog 27 | No | Baseline | 22 | 60 | **137** | 3 |
|  |  | 3-month | 51 | 46 | 105 | 6 |
|  |  | 6-month | 62 | 32 | 73 | 6 |
|  |  | 9-month | --- | --- | --- | --- |
|  |  | 12-month | --- | --- | --- | --- |
| Dog 28 | Yes | Baseline | 47 | 39 | **234** | 6 |
|  |  | 3-month | 45 | 45 | 105 | 10 |
|  |  | 6-month | 41 | 32 | 66 | 7 |
|  |  | 9-month | 33 | 25 | 74 | 2 |
|  |  | 12-month | 35 | 30 | 27 | 1 |
| Dog 29 | No | Baseline | 17 | 29 | 33 | 3 |
|  |  | 3-month | 33 | 33 | 28 | 8 |
|  |  | 6-month | --- | --- | --- | --- |
|  |  | 9-month | --- | --- | --- | --- |
|  |  | 12-month | --- | --- | --- | --- |
| Dog 30 | No | Baseline | 20 | 21 | 50 | 2 |
|  |  | 3-month | 32 | 28 | 23 | 2 |
|  |  | 6-month | 32 | 28 | 70 | 2 |
|  |  | 9-month | --- | --- | --- | --- |
|  |  | 12-month | --- | --- | --- | --- |
| Dog 31 | No | Baseline | 25 | 21 | 100 | 4 |
|  |  | 3-month | 43 | 25 | **230** | 5 |
|  |  | 6-month | --- | --- | --- | --- |
|  |  | 9-month | --- | --- | --- | --- |
|  |  | 12-month | --- | --- | --- | --- |
| Dog 32 | No | Baseline | 37 | 31 | 99 | 1 |
|  |  | 3-month | 66 | 42 | **236** | 5 |
|  |  | 6-month | --- | --- | --- | --- |
|  |  | 9-month | --- | --- | --- | --- |
|  |  | 12-month | --- | --- | --- | --- |

ALT, alanine aminotransferase; ALP, alkaline phosphatase; GGT, gamma-glutamyl transferase; AST, aspartate aminotransferase
