# Supplementary material for: Serial evaluation of liver enzyme activities in dogs with pulmonary coccidioidomycosis administered per os fluconazole
Source: Front Vet Sci. 2024 Jul 3;11:1402572. doi: 10.3389/fvets.2024.1402572 (PMC11417468; doi:10.3389/fvets.2024.1402572)
Supplement: Supplementary file 2 [file Table_2.DOCX]

**Supplemental Table 2.** Association between baseline variables and the development of an elevation in alanine transaminase (ALT) activity on at least 1 evaluation in 32 dogs with pulmonary coccidioidomycosis after administration of per os fluconazole.

| **Variable** | **Odds ratio** | **95% CI** | **P-value** |
| --- | --- | --- | --- |
| **Age (years)** | 1.00 | 0.75-1.35 | > 0.9 |
| **Weight (kg)** | 0.94 | 0.83-1.06 | 0.3 |
| **Sex** |  |  |  |
| Male | — | — |  |
| Female | 4.20 | 0.49-65.4 | 0.3 |
| **Neutered** |  |  |  |
| Intact | — | — |  |
| Neutered | 0.94 | 0.08-12.3 | > 0.9 |
| **Prednisone administration** |  |  |  |
| No Prednisone | — | — |  |
| Prednisone | 0.69 | 0.05-5.79 | 0.7 |
| **Fluconazole dose (mg/kg/day)** | 1.03 | 0.83-1.33 | 0.8 |
| **Duration of fluconazole administration (days)** | 1.01 | 1.00-1.02 | 0.2 |

Kg, kilogram; mg, milligram; CI, confidence interval
