# Supplementary material for: Serial evaluation of liver enzyme activities in dogs with pulmonary coccidioidomycosis administered per os fluconazole
Source: Front Vet Sci. 2024 Jul 3;11:1402572. doi: 10.3389/fvets.2024.1402572 (PMC11417468; doi:10.3389/fvets.2024.1402572)
Supplement: Supplementary file 3 [file Table_3.DOCX]

**Supplemental Table 3.** Association between baseline variables and the development of an elevation in alkaline phosphatase (ALP) activity on at least 1 evaluation in 32 dogs with pulmonary coccidioidomycosis after administration of per os fluconazole.

| **Variable** | **Odds ratio** | **95% CI** | **P-value** |
| --- | --- | --- | --- |
| **Age (year)** | 1.13 | 0.86-1.53 | 0.4 |
| **Weight (kg)** | 0.97 | 0.88-1.06 | 0.5 |
| **Sex** |  |  |  |
| Male | — | — |  |
| Female | 0.68 | 0.09-5.51 | 0.7 |
| **Neutered** |  |  |  |
| Intact | — | — |  |
| Neutered | 1.06 | 0.11-11.4 | >0.9 |
| **Prednisone administration** |  |  |  |
| No Prednisone | — | — |  |
| Prednisone | 0.16 | 0.01-1.31 | 0.13 |
| **Fluconazole dose (mg/kg/day)** | 1.13 | 0.90-1.53 | 0.3 |
| **Duration of fluconazole administration (mg/kg/day)** | 1.00 | 0.99-1.01 | 0.6 |

Kg, kilogram; CI, confidence interval; mg, milligram
